# Supplementary material for: Investigating the Effect of Surface Hydrophilicity on the Destiny of PLGA-Poloxamer Nanoparticles in an In Vivo Animal Model
Source: Int J Mol Sci. 2023 Sep 25;24(19):14523. doi: 10.3390/ijms241914523 (PMC10572154; doi:10.3390/ijms241914523)
Supplement: Supplementary file 1 [file ijms-24-14523-s001.zip › ijms-2562123-supplementary.pdf]

Supplementary information

Table S1. Entrapment efficiency and release results

**Sample**	**P NPs ng mL^{-1}**	**PP NPs ng mL^{-1}**
Entrapment efficiency		
Post first centrifugation	1307.64	1032.44
Post second centrifugation	821.25	547.00
Post third centrifugation	819.07	513.46
Release		
Mean 1 h	726.39 ± 375.51	37.14 ± 27.57
Mean 4 h	700.55 ± 304.54	36.39 ± 7.46
